# Sleep as the Hidden Cost of mWork: Unpacking the Roles of Job Stress, Gender, and Number of Children

**DOI:** 10.3390/bs15070857

**Published:** 2025-06-25

**Authors:** Woo-Sung Choi, Hee Jin Kim, Sung-woo Cho, Seung-Wan Kang, Hyeran Choi

**Affiliations:** 1 College of Business, Gachon University, Seongnam 13120, Republic of Korea; wschoi2@gachon.ac.kr (W.-S.C.); heejinkim@gachon.ac.kr (H.J.K.); sungwoo@gachon.ac.kr (S.-w.C.); 2 College of Business & Economics, Chung-Ang University, Seoul 06974, Republic of Korea

**Keywords:** mWork, job stress, sleep deprivation, gender, number of children, conservation of resources (COR) theory

## Abstract

The widespread adoption of mobile work, driven by advancements in information and communication technology, has increasingly blurred the boundaries between work and personal life. This phenomenon can increase job stress, potentially leading to sleep deprivation, which affects not only employees’ health and well-being but also organizational performance. Grounded in Conservation of Resources theory, this study examines the pathway through which mWork contributes to sleep deprivation, focusing on the mediating role of job stress, and investigates the moderating effects of gender and number of children on this relationship. Data were collected using a stratified random sampling method across three waves with 4-week intervals, involving 325 employees in South Korea engaged in diverse occupations, including the administrative, technical, service, and sales sectors. The findings reveal that mWork increases sleep deprivation through the mediation of job stress. Furthermore, the relationship between mWork and job stress was found to vary depending on gender and number of children, with stronger moderating effects observed among women and employees with children. This study underscores the need for organizations to develop tailored management strategies that address the unique challenges posed by mWork, taking particular note of employees’ gender and family responsibilities. By mitigating the negative effects of mWork on job stress and sleep deprivation, organizations can enhance employee well-being and promote sustainable long-term performance.

## 1. Introduction

The rapid digitalization of today’s workplace has brought transformative changes in work environments. Advances in information and communication technology (ICT) have enabled the widespread adoption of mobile work, allowing employees to perform job tasks beyond traditional working hours and outside conventional office settings (Choi et al., 2024a; Ferguson et al., 2016). This study defines mWork as the use of ICT to carry out work-related tasks outside standard working hours and beyond the physical boundaries of the office or home environment. While mWork offers temporal flexibility, it can also blur the boundaries between work and personal life, potentially leading to increased job stress and sleep deprivation (Putri & Amran, 2021; Eurofound, 2020a, 2020b). Additionally, mWork often emerges as a key driver of job stress by eroding the separation between personal and professional domains (Ferguson et al., 2016).

Job stress arises when there is a mismatch between job demands and an individual’s capabilities, resulting in emotional exhaustion caused by tension, worry, or frustration. Such stress can manifest as negative emotions, including anxiety, frustration, and decreased organizational commitment, ultimately undermining employee performance and organizational outcomes (Bakker & De Vries, 2021; Crawford et al., 2010; Jamil et al., 2023). By depleting employees’ recovery resources, mWork can exacerbate job stress, which in turn increases the likelihood of sleep deprivation (Hobfoll et al., 2018).

Research on the relationship between job stress and sleep quality has consistently shown that job stress impairs sleep, which subsequently hinders job performance (Barnes et al., 2017). Sleep deprivation, in particular, can lead to fatigue and cognitive impairment, making employees more vulnerable to stress, thus creating a vicious cycle (Zacher, 2015). These dynamics can be explained through the lens of the Conservation of Resources (COR) theory, which posits that job stress leads to the continual loss of psychological and physical resources, further intensifying resource depletion through sleep deprivation (Stewart & Arora, 2019).

To better understand the relationship between mWork and job stress, it is essential to examine how individuals’ resources are consumed. When the boundaries between work and personal life become increasingly blurred, work-related demands persistently deplete psychological and emotional resources, culminating in elevated stress levels (Baker et al., 2022; Dolsen et al., 2019; Edú-Valsania et al., 2022; Tarafdar et al., 2019). The inability to replenish recovery resources exacerbates resource loss, thereby intensifying stress (Bakker & Demerouti, 2017; Hobfoll et al., 2018). These effects are particularly pronounced among female employees and employees with children, who are more likely to experience the negative consequences of mWork (Sasidharan, 2022; Topsakal & Irmak, 2023).

This study focuses on the moderating roles of gender and number of children in the relationship between mWork and job stress. Although previous studies have explored the relationships among mWork, job stress, and sleep deprivation, they have primarily focused on simple mediation effects and rarely examined the moderating roles of individual and family characteristics. Notably, Dolce et al. (2024) and Ciaccio et al. (2021) specifically called for research that incorporates moderators such as gender and family variables, highlighting a clear gap in the literature. By empirically testing the moderating effects of both gender and number of children, this study directly addresses these limitations and advances our understanding of how mWork environments affect employee well-being. Previous studies have examined the significant impact of the number of children on job stress levels and sleep quality. However, few studies have integratively analyzed the relationships among mWork, job stress, sleep deprivation, and individual characteristics such as gender and number of children (Schieman et al., 2021).

By empirically examining the differential effects of mWork based on these individual characteristics, this study aims to provide a deeper understanding of the relationships among mWork, job stress, and sleep deprivation. In doing so, it extends the application of COR theory by uncovering the moderating effects of personal characteristics that have been underexplored in prior research. Moreover, it offers important implications for organizations and managerial practice by highlighting the need for tailored support strategies to alleviate the adverse effects of mWork. Incorporating individual characteristics, such as gender and number of children, it provides valuable policy recommendations aimed at reducing job stress and enhancing sleep quality, ultimately promoting employee well-being and organizational performance.

## 2. Theoretical Background and Hypothesis

### 2.1. Conservation of Resources Theory

The COR theory asserts that individuals prioritize the acquisition, preservation, and protection of resources they perceive as valuable (Hobfoll et al., 2018). This tendency becomes particularly evident when resources are threatened or depleted, resulting in increased stress, decreased job satisfaction, and a deep sense of loss. To address these challenges, individuals actively work to minimize resource losses, whether cognitive or material, and to restore depleted resources. Resources are broadly defined as tangible assets, personal states, and favorable conditions that hold importance for individuals.

From this perspective, adequate sleep is considered a fundamental personal resource. Sleep functions as a vital psychological and physiological resource for employees, and insufficient sleep can accelerate resource depletion and increase vulnerability to stress and maladaptive workplace behaviors (Marcatto et al., 2025). Marcatto et al. (2025) empirically demonstrated that sleep impairment mediates the relationship between work-related stress and negative organizational outcomes, while Sun et al. (2024) found that job stress significantly lowers sleep quality, leading to health-related productivity loss. These findings highlight the importance of adequate sleep as a core resource in managing occupational stress and sustaining employee well-being.

Sleep deprivation disrupts not only job performance but also organizational effectiveness. The relationship between sleep deprivation and its antecedents can be explained within the COR framework. High job demand, such as that associated with mWork, leads to resource depletion and loss, which in turn heightens job stress. Elevated job stress can subsequently result in sleep deprivation. Therefore, organizations must develop strategies and policies to help employees build and maintain diverse resources (Choi et al., 2024b). This study applies COR theory to investigate how mWork, job stress, gender, and the number of children influence sleep deprivation as antecedent factors.

### 2.2. mWork and Sleep Deprivation

Ferguson et al. (2016) define mWork as “the technology interface or use of a mobile device to engage in work activities during family time,” and emphasize its significance by stating that “we focus on mWork in that it allows the user to attend to work issues that transcend the time and location constraints of the physical workplace and the typical workday” (p. 520). Kauffeld et al. (2022) describe mWork as work performed outside the traditional office setting that is enabled by ICT, allowing employees to carry out their tasks anytime and anywhere.

While mWork offers advantages such as increased productivity and greater flexibility, it can also lead to various negative outcomes (Golden & Eddleston, 2020). Individuals strive to protect and conserve resources they value, and when these resources are threatened or depleted, they experience stress (Hobfoll et al., 2018). mWork extends the connection to work beyond normal hours, consuming psychological and physical resources, which can eventually lead to a decline in sleep quality (Garefelt et al., 2020; Van Laethem et al., 2018; Zagaria et al., 2023).

Prior research has consistently highlighted the connection between mWork and sleep deprivation. Previous studies reported that using devices like smartphones for work diminishes individuals’ ability to mentally disengage from their job, which adversely affects their sleep (de Sá et al., 2023; Derks et al., 2015, 2016; Kheirinejad et al., 2023; Kumar et al., 2025). Similarly, studies have shown that engaging with work-related devices outside of working hours disrupts recovery, contributing to sleep deprivation and heightened fatigue (Xie et al., 2018). Furthermore, checking work emails disrupts sleep quality, as it diminishes the separation between work and personal life; greater work–home boundary ambiguity is associated with lower sleep quality and higher levels of fatigue (Barber & Santuzzi, 2015; Park et al., 2018).

These findings highlight how mWork can cause sustained psychological activation during non-work hours, contributing to sleep deprivation (Bolino et al., 2021). mWork accelerates resource depletion and limits opportunities for recovery, resulting in poorer sleep quality and quantity. For these reasons, mWork is increasingly recognized as a key factor contributing to sleep deprivation. Therefore, we propose the following hypothesis:

**Hypothesis** **1.** *mWork has a positive relationship with sleep deprivation, meaning that higher levels of mWork are associated with greater sleep deprivation.*

### 2.3. The Mediating Role of Job Stress

mWork may increase job stress by restricting employees’ opportunities for recovery through the use of their personal time. Elevated job stress accelerates resource depletion, negatively affecting sleep quality and overall health (Åkerstedt et al., 2002; Hasin et al., 2023; Mohammadinejad et al., 2025; Sun et al., 2024). The use of work-related mobile devices increases job stress, which in turn leads to fatigue and sleep deprivation (Åkerstedt et al., 2011; Derks et al., 2016).

Job stress can impair sleep quality by disrupting the balance between work demands and recovery (Hetland et al., 2022; Sonnentag & Fritz, 2015). The depletion of resources limits opportunities for recovery, resulting in fatigue and sleep disturbances (Asadullah et al., 2024; Boncoeur, 2021; Hobfoll et al., 2018). Furthermore, job stress induces psychological arousal, which can hinder the initiation and maintenance of sleep (Barnes et al., 2013). Higher levels of job stress are thus associated with poorer sleep quality, highlighting the significant impact of work-related stressors on sleep (Xie et al., 2018).

Given these findings, job stress is likely to mediate the relationship between mWork and sleep deprivation. Specifically, job stress acts as a critical mechanism that explains how mWork depletes resources and contributes to sleep deprivation. Therefore, we propose the following hypothesis:

**Hypothesis** **2.** *Job stress mediates the relationship between mWork and sleep deprivation. In other words, mWork increases job stress, and elevated job stress subsequently leads to greater sleep deprivation.*

### 2.4. The Moderating Role of Gender

Gender may serve as a critical moderator in the relationship between mWork and job stress. Prior research suggests that women are more likely than men to struggle with maintaining boundaries between work and home life, which may make them more susceptible to stress arising from mWork (Dahlberg et al., 2021; Eddleston & Powell, 2012; Rodríguez-Domínguez et al., 2022). Additionally, societal expectations and traditional gender roles often place women under additional burdens, such as household responsibilities and caregiving, further exacerbating job stress (Li et al., 2022; Matud, 2004). Women may be more vulnerable to resource depletion, which could lead to heightened stress in mWork contexts (Hobfoll et al., 2018).

Previous studies have highlighted gender differences in stress responses, suggesting that women may be more sensitive to emotional stressors compared to men (Bakker et al., 2008; Cholankeril et al., 2023). These findings support the notion that the intensity of job stress caused by mWork may vary by gender. Furthermore, gender influences the strategies employed for preserving psychological and physical resources, with women often being more affected by resource depletion than men (Eagly, 2007; Pergelova et al., 2025).

It is also reported that gender moderates the relationship between job stress and its health-related outcomes, indicating that the resource depletion caused by mWork may have a more pronounced impact on women (Pergelova et al., 2025; Xie et al., 2018). These findings underscore the importance of considering gender as a significant moderator in understanding the link between mWork and job stress. Therefore, we propose the following hypothesis:

**Hypothesis** **3.** *Gender moderates the relationship between mWork and job stress, such that the positive effect of mWork on job stress is stronger for women than for men.*

### 2.5. The Moderating Role of the Number of Children

In addition to gender, the number of children an employee has may serve as a critical moderating factor in the relationship between mWork and job stress. Both gender and number of children are relatively stable sociodemographic characteristics that act as structural, objective variables influencing individuals’ baseline resource conditions. In contrast, constructs such as work–family conflict and caregiving responsibility—while related—are subjective situational outcomes shaped by personal experiences. Gender and number of children serve as contextual indicators of resource vulnerability, reflecting individuals’ differential exposure to competing demands and constraints on personal resources, whereas work–family conflict and caregiving burden are better understood as manifestations of resource depletion or mediators in the stress process (Allen et al., 2014; Bakker & Demerouti, 2007; Thébaud et al., 2024; Yenn et al., 2024).

Although this study does not directly examine work–family conflict, given its role as a mediating variable, its higher prevalence among employees with children offers indirect support for treating the number of children as a structural moderator of job stress. Specifically, employees with children are more likely to experience heightened work–family role conflict, which can exacerbate job stress (Byron, 2005). Job stress, compounded by familial responsibilities and obligations, accelerates resource depletion, a dynamic that can be better understood within the framework of COR theory. Employees with children face additional demands, such as childcare and household responsibilities during non-working hours, which may intensify the process by which mWork contributes to job stress (Geurts et al., 2005; Matthews et al., 2022).

The dual roles of work and family are particularly burdensome for employees with children, often resulting in greater stress (Allen et al., 2014). This suggests that employees with children may experience higher levels of job stress due to mWork. Furthermore, the lack of time for recovery among employees with children increases the likelihood that mWork will lead to sleep deprivation (Bianchi & Milkie, 2010). These findings indicate that the number of children may moderate the impact of resource depletion on job stress (Fan & Moen, 2024; Martire et al., 2004; Schnettler et al., 2025), with the effects becoming more pronounced as the number of children increases.

Employees with children may exhibit stronger stress responses when resources are significantly depleted, positioning the number of children as an important moderating factor in the relationship between mWork, job stress, and sleep deprivation (Graham et al., 2021; Neff & Faso, 2015; Shockley et al., 2021). These findings suggest that the presence of children amplifies the stress caused by mWork, increasing the likelihood that job stress will escalate in such circumstances.

**Hypothesis** **4.** *The number of children moderates the relationship between mWork and job stress, such that the positive association between mWork and job stress is amplified as the number of children increases.*

**Figure 1:**
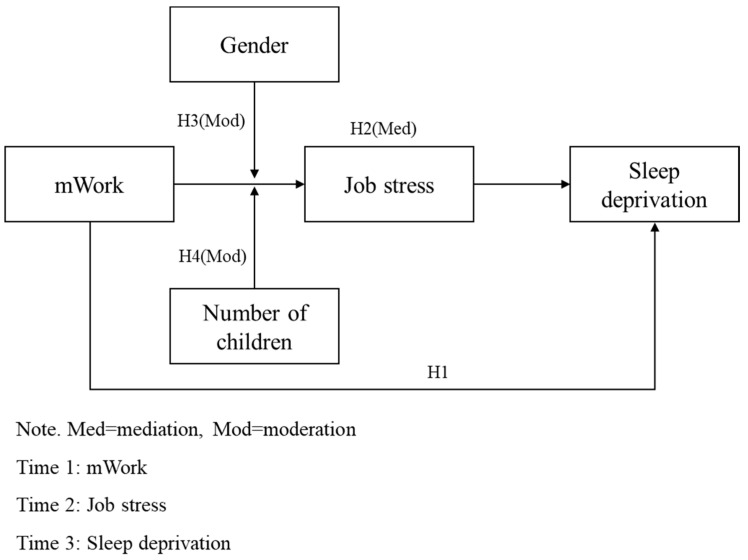
Research Model.

The research model is illustrated in Figure 1.

## 3. Method

### 3.1. Sampling Procedure

To address potential common method bias (CMB) inherent in cross-sectional surveys, this study adopted a three-wave design, measuring variables at three separate intervals set 1 month apart. This decision was based on prior research, which suggests that a 1-month interval is sufficient to capture meaningful changes in work experiences and psychological states, while also reducing CMB and recall bias (Di Blasi et al., 2022; Fuller et al., 2016). Moreover, similar longitudinal studies in organizational behavior have adopted a 1-month interval, further supporting the validity of this choice of timing (Di Blasi et al., 2022).

Data were collected through a professional online survey company, a subsidiary of a globally recognized marketing research organization known for reliable and robust data collection. We used a stratified random sampling method to ensure that the sample accurately represented the distribution of Korean employees by gender and age. Inclusion criteria required participants to be adults aged 20 or older who were currently employed; minors and non-working individuals were excluded. This approach ensured the acquisition of high-quality, dependable data, thereby enhancing the validity and reliability of the study’s outcomes.

Participants consisted of South Korean professionals employed in various industries, all of whom reported to a supervisor at their respective workplaces. Respondents were selected using a stratified random sampling method. Before participating in the survey, participants were provided with detailed information about the study’s purpose, procedures, and their right to withdraw at any time, and the potential risks and benefits of participation. Informed consent was obtained from all participants, and only those who agreed to participate were included in the study. All data were anonymized and securely stored in a separate electronic database under the principal investigator’s supervision.

The first survey was sent to 1200 individuals, yielding 672 valid responses after excluding unreliable data, for a response rate of 56%. One month later, the second survey was distributed to 672 respondents, of whom 450 provided valid responses, reflecting a retention rate of 37.5% from the original sample. Another month later, the third survey was sent to the 450 participants who had completed the first two surveys, resulting in 325 valid responses after filtering out unreliable data. This final wave corresponded to a retention rate of 27.1% from the initial sample of 1200 participants (Appendix A).

Of the 325 respondents, 70.5% were employed in office roles, 11.7% in manufacturing, 10.5% in service positions, and 7.3% in sales or other occupations. The sample was nearly evenly divided by gender, with 50.2% identifying as female and 49.8% as male. The average age of participants was 41.8 years (SD = 11). Regarding educational background, 53.2% held a 4-year bachelor’s degree, 20.9% had a 2-year associate degree, 18.8% had completed high school, 5.6% held a master’s degree, and 1.5% held a doctoral degree. The average tenure at their current workplace was 7.7 years (SD = 6.8). Additionally, 50.5% reported having no children, 17.5% reported having one child, 28.9% reported having two children, 2.5% reported having three children, and the remaining 0.6% reported having four or more children.

### 3.2. Measurement

The variables in this study were measured using a 5-point Likert scale (1 = strongly disagree, 5 = strongly agree). The original survey, developed in English, was translated into Korean and subsequently reviewed and refined by linguistic experts. To ensure the accuracy of the translation, the Korean version was back-translated into English, allowing for a comparison of linguistic and semantic consistency with the original version. The back-translation process validated the accuracy and conceptual equivalence of the translation (Appendix B).

#### 3.2.1. mWork

The frequency of engaging in mWork outside regular working hours was measured using three items developed by Ferguson et al. (2016). An example item included, “I frequently use my smartphone or laptop to work after office hours or on weekends.” Cronbach’s alpha coefficient for this scale was 0.92.

#### 3.2.2. Job Stress

Job stress was measured using four items from Keller (1984). These items capture various aspects of job-related stress. An example item included, “I often feel frustrated due to my work.” Cronbach’s alpha for this scale was 0.85.

#### 3.2.3. Sleep Deprivation

Sleep deprivation was measured using four items developed by Barnes et al. (2017), designed to capture poor sleep quality and quantity. An example item included, “I wake up feeling tired and exhausted, even after sleeping my usual amount.” The scale demonstrated strong reliability, with a Cronbach’s alpha of 0.83.

### 3.3. Common Method Bias

To minimize the risk of CMB, the survey was conducted in three waves at different time intervals. In addition to this procedural remedy, two statistical techniques were employed to assess the extent of CMB. First, Harman’s single-factor test was conducted using all items (n = 325); the first factor accounted for only 25.3% of the total variance, suggesting that CMB was not a serious concern (Fuller et al., 2016). Second, a marker variable technique was applied by comparing the baseline model and the marker model. The results showed that there were no significant differences in model fit indices or key structural paths between the two models, further indicating that the findings were unlikely to be substantially influenced by CMB (Podsakoff et al., 2003).

### 3.4. Research Strategy

To verify the validity of the research model, confirmatory factor analysis (CFA) was conducted. Hypotheses were tested using hierarchical regression analysis employing STATA 17.0 (Stata Corp., College Station, TX, USA). Mediation effects were examined using the bootstrapping technique as recommended by Hayes (2018).

## 4. Result

### 4.1. Descriptive Statistics and Correlation Analysis

Table 1 presents the means, standard deviations, correlations, and Cronbach’s α values for the study variables. Significant correlations consistent with the study hypotheses were observed among the variables.

### 4.2. Confirmatory Factor Analysis

Table 2 presents the results of the CFA conducted to evaluate the construct validity of the study variables. The chi-square ratio to degrees of freedom was 1.75, well below the acceptable threshold of 3.00. Additionally, the Comparative Fit Index (CFI) and Tucker–Lewis Index (TLI) achieved values of 0.97 and 0.96, both exceeding the recommended benchmark of 0.95 (Hair et al., 2023). The root mean square error of approximation (RMSEA) was 0.05, meeting both the acceptable upper limit of 0.08 and the preferred standard of 0.05 (Hair et al., 2023). Based on these fit indices, the hypothesized three-factor model demonstrated excellent fit. Moreover, when compared to two alternative models, the three-factor structure emerged as the most suitable representation of the data.

### 4.3. Hypothesis Testing

Hypotheses 1, 3, and 4 were tested using hierarchical multiple regression analysis, while Hypothesis 2 was examined using the bootstrapping technique. The results for Hypothesis 1, as presented in Model 5 of Table 3, revealed a significant positive relationship between mWork and sleep deprivation. Furthermore, Model 5 demonstrated greater explanatory power compared to Model 4, providing support for Hypothesis 1.

Hierarchical regression models were constructed to test the main and moderating effects proposed in Hypotheses 1, 3, and 4. Model 1 included only the control variables. Model 5 introduced mWork to assess its direct association with job stress and sleep deprivation, thereby addressing Hypothesis 1. Model 3 incorporated interaction terms between mWork and gender, and between mWork and number of children, to assess the moderating roles of these variables as specified in Hypotheses 3 and 4. This modeling approach enables a systematic evaluation of the incremental explanatory power of each predictor and interaction term, providing a clear framework for testing the hypothesized relationships.

Hypothesis 2 was tested through an analysis of indirect effects using 10,000 bootstrapped iterations. As detailed in Table 4, the 95% confidence interval (95% CI = [0.01, 0.07]) did not include zero, thereby confirming support for Hypothesis 2.

**Figure 2:**
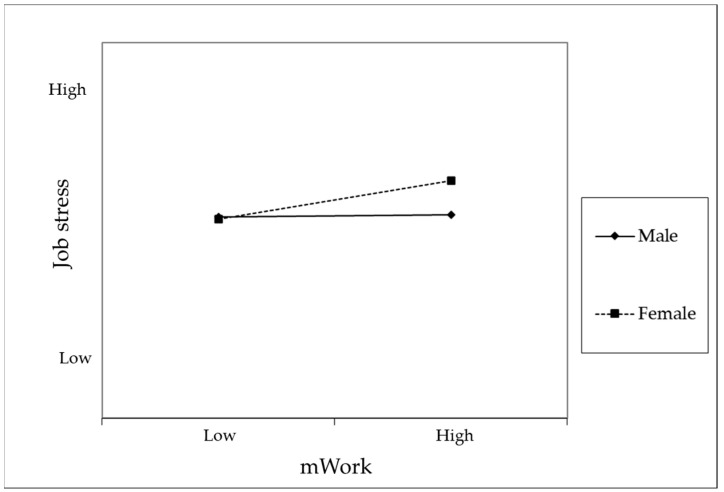
Moderating Effect of Gender on the Relationship Between mWork and Sleep Deprivation.

Hypothesis 3 proposed that the relationship between mWork and job stress would be moderated by gender. As shown in Model 3 of Table 3, the interaction term between mWork and gender had a significant effect on job stress. Furthermore, Model 3 demonstrated a significant improvement in explanatory power compared to Model 2. A subsequent simple slope analysis (see Figure 2) revealed that the relationship between mWork and job stress was significant for women but not for men. These findings confirm the presence of a moderating effect of gender.

**Figure 3:**
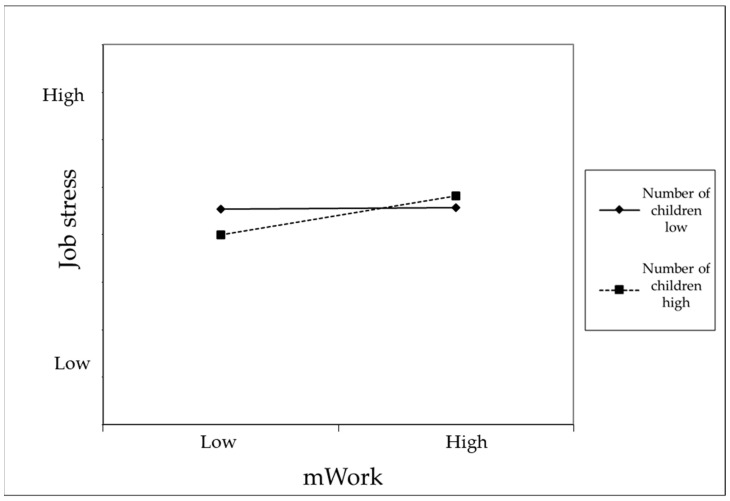
Moderating Effect of the Number of Children on the Relationship Between mWork and Sleep Deprivation.

Hypothesis 4 proposed that the relationship between mWork and job stress would be moderated by the number of children. As shown in Model 3 of Table 3, the interaction term between mWork and the number of children significantly influenced job stress. Additionally, Model 3 demonstrated a statistically significant improvement in explanatory power compared to Model 2. To examine the moderating effect of the number of children on the relationship between mWork and job stress, we mean-centered the number of children variable prior to analysis, following the recommendations of Aiken and West (1991). Simple slopes were estimated at one standard deviation below (−1 SD) and above (+1 SD) the mean of the number of children variable. Simple slope analysis (see Figure 3) revealed that the relationship between mWork and job stress was significant for individuals with more children, but not for those with fewer children. These findings confirm the moderating effect of the number of children.

**Figure 4:**
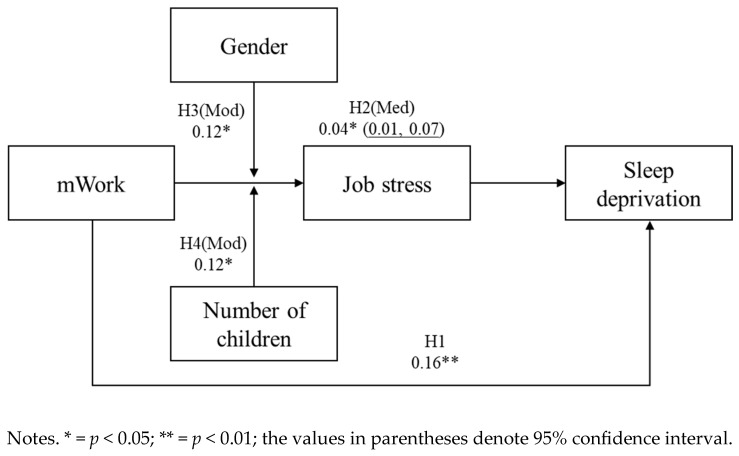
Regression and bootstrapped indirect effect test results.

The verification of hypotheses and relationships between research variables, established through regression analysis and bootstrapping mediation analysis, is depicted in Figure 4.

## 5. Discussion

### 5.1. Theoretical Contribution

This study makes significant theoretical contributions by empirically analyzing the impact of mWork on job stress and sleep deprivation through the lens of COR theory. First, the study extends the understanding of the negative consequences of mWork. While previous research has primarily highlighted the positive aspects of mWork, such as providing flexible work arrangements and enhancing productivity and efficiency, this study demonstrates that mWork can also lead to adverse outcomes, including increased job stress and sleep deprivation. By applying COR theory to illustrate the mechanisms of resource depletion and stress generation, this research deepens the academic discourse on the potential drawbacks of mWork.

Second, this study clarifies the causal pathway between mWork and sleep deprivation by identifying the mediating role of job stress. The findings reveal that the process of resource depletion is not unidimensional; instead, job stress acts as an intermediary mechanism, linking mWork to the outcome of sleep deprivation. This contributes to the theoretical development of COR theory by illustrating that job stress serves as a critical stage in the progression from resource loss caused by mWork to physical consequences such as poor sleep. Furthermore, it highlights that job stress not only affects job performance and satisfaction but can also have significant implications for employees’ physical resources, such as sleep quality.

This mediating pathway is consistent with prior research showing that ICT-based work increases psychological fatigue and ultimately impairs sleep quality (Derks et al., 2014). Additionally, job stress has been identified as a primary driver of sleep deprivation, with detrimental effects on individual performance and long-term health outcomes (Barnes et al., 2012). By systematically explaining the mechanisms linking mWork, job stress, and sleep deprivation, this study expands on these earlier findings.

Third, the study provides insights into differential responses to mWork-induced job stress by examining the moderating effects of gender and number of children. While prior research has discussed the impact of gender and parental responsibilities on job stress (Lott et al., 2022; Schieman et al., 2021), this study empirically demonstrates that these factors moderate the relationship between mWork and job stress.

The findings suggest that female employees and those with children may experience relatively higher levels of job stress due to mWork, although the moderation effects were modest. The present model explains only 9% of the variance in sleep deprivation, indicating that other factors—such as work intensity or organizational culture—may also play important roles in this relationship. Although the effect sizes are small, they nonetheless offer valuable theoretical insights; however, caution is warranted when interpreting the practical implications of these findings. This underscores the critical role of gender and the number of children in the processes of resource conservation and depletion, offering a more nuanced application of COR theory. Notably, the stronger moderating effect observed for women provides a foundation for further understanding gender differences in responses to job stress, advancing scholarly discussions in this area.

Last, this study highlights the role of the number of children in moderating the relationship between mWork and job stress, shedding light on how family structure influences stress levels. Employees with children experience greater stress due to the boundary-blurring effects of mWork. This suggests that parents encounter greater challenges in securing recovery time, which exacerbates the process by which job stress contributes to sleep deprivation. The findings empirically demonstrate that the dynamics of resource loss and conservation, as outlined in COR theory, can vary based on an individual’s family background. Furthermore, our model includes gender and number of children as stable sociodemographic factors that influence a person’s vulnerability to resource loss. These differ from experience-based factors, such as work–family conflict, which may vary depending on the situation. This suggests that both fixed structural factors and dynamic experiential factors should be considered together to gain a better understanding of how job stress develops.

The theoretical contributions of this study lie in its in-depth exploration of the negative effects of mWork on resource depletion and stress generation. By identifying job stress as a pathway leading to physical outcomes such as sleep deprivation, the study broadens the empirical applicability of COR theory. These findings serve as a foundational resource for future research on mWork, emphasizing the importance of individual characteristics and resource management in mitigating its negative consequences.

### 5.2. Practical Implications

The findings of this study offer several practical implications for organizational managers and policymakers. First, organizations should implement appropriate boundary-setting practices and employee welfare programs to mitigate the negative effects of mWork. The results indicate that mWork can lead to increased job stress and sleep deprivation. For instance, initiatives such as flexible work arrangements, informal counseling sessions, or stress management workshops can support employees in managing job stress and conserving psychological resources.

Second, considering the mediating role of job stress, managers should encourage employees to have adequate opportunities for recovery during non-working hours. This aligns with the resource recovery process emphasized in COR theory. Organizations should establish ICT usage guidelines to ensure employees can rest and recharge after work. For example, policies could restrict sending emails or messages outside working hours or clearly delineate work and non-work hours to minimize intrusions.

Third, differentiated support should be tailored to various employee groups, reflecting the moderating effects of gender and number of children. Women and employees with children are more likely to experience heightened stress from mWork, making it essential to offer targeted support programs for these groups. Organizations can address these issues by fostering family-friendly work environments and introducing flexible work hours and childcare support programs for working parents.

Fourth, this study shows that stable factors, such as gender and the number of children someone has, can strongly influence how employees experience stress from mWork. Instead of focusing only on individual feelings or short-term situations, we took a step back to look at longer-term patterns that affect many people. Practically speaking, this means organizations should consider these differences when making policies. For instance, adjusting workloads or providing supportive amenities like a rest area that directly address the unique challenges faced by women with multiple children. While these steps are unlikely to solve every problem, they are important for a healthier and more equitable work environment.

Last, greater effort is needed from organizational leaders to create family-friendly workplaces and promote work-life balance. This is particularly relevant in addressing broader societal challenges, such as low birth rates in countries like South Korea. Managers should recognize that the negative effects of mWork are more pronounced among female employees and incorporate this awareness into work allocation and policy design. For female employees with children, societal expectations tied to gender roles can amplify the impact of mWork. For these employees, managers should encourage a focus on productivity during regular working hours and avoid contacting them during evenings or weekends.

While our findings indicate that the positive association between mWork and job stress is more pronounced among women and further intensified with an increasing number of children, it is essential to interpret these results with careful nuance. These patterns should not be construed as evidence of inherent vulnerabilities or deficiencies among particular demographic groups. Rather, they reflect the broader structural and societal conditions—such as unequal caregiving expectations and persistent gender norms—that disproportionately shape the lived experiences of women and working parents (Lahaie et al., 2013). Accordingly, we urge that these findings be understood not as grounds for differential treatment in hiring, promotion, or evaluation, but as a compelling call for organizations to adopt more equitable and supportive workplace practices (e.g., Cavagnis et al., 2023; Chen, 2016). The moderating effects observed in this study identify critical areas where targeted interventions are urgently needed to reduce the negative consequences of mWork and to promote inclusive well-being across diverse employee groups.

To this end, organizations should prioritize the development of comprehensive support systems that directly address the unique challenges faced by women and employees with caregiving responsibilities (cf., Bohlmann & Zacher, 2019; Turgeman-Lupo et al., 2020). These may include policies that foster temporal flexibility, provide access to childcare support, establish clear boundaries between work and personal time, and expand access to psychological resources (cf., Blithe, 2023; Conroy, 2019; Laksmi et al., 2024). Implementing such measures is not only ethically imperative but also instrumental in sustaining employee well-being and performance in increasingly mobile and boundaryless work environments. Ultimately, our findings underscore the enduring influence of gendered societal expectations and the additional burdens they place on individuals navigating both professional and domestic spheres. Addressing these challenges requires organizational commitment to structural change, enabling all employees—regardless of gender or family status—to thrive in the modern workplace.

### 5.3. Research Limitations and Future Research Directions

While this study provides valuable insights for both scholars and practitioners, it has several limitations that warrant consideration in future research.

First, the study’s sampling method and scope present certain constraints. Although data were collected through a three-wave time-lagged survey design, the measurement of variables was limited to specific points in time, reflecting the cross-sectional nature of the research. Future studies should consider employing longitudinal designs to establish stronger causal inferences. Additionally, as the measurement of variables relied on data from a single source, there is a possibility of CMB. Although CFA confirmed the distinctiveness of the variables, future research should address this limitation by incorporating data from multiple sources.

Second, as the survey targeted South Korean employees, the findings may be influenced by cultural factors specific to the Korean context. Thus, caution should be exercised when generalizing these results to other cultural settings. Future research could explore the effects of mWork on sleep deprivation across diverse cultural contexts, offering opportunities for comparative analysis and new insights.

Third, this study employed COR theory to explain the mechanisms through which mWork, job stress, gender, and the number of children influence sleep deprivation. However, integrating or proposing alternative theoretical frameworks could yield additional insights into the relationship between mWork and sleep deprivation.

Fourth, while this study focused on gender and the number of children as moderators, mWork is inherently a variable likely to be influenced by various conditions and situational factors. Looking forward, future research might explore how other organizational factors, such as different work arrangements or compensation systems, influence the relationship between mWork and stress. It would also be valuable to investigate how stable factors like gender and the number of children interact with more dynamic, experience-based factors such as work–family conflict. For example, work–family conflict may function as an intervening mechanism linking these background characteristics to stress-related outcomes. Using advanced methods like multilevel moderated mediation or testing complex interactions (for instance, gender × number of children × work–family conflict) could offer a richer understanding of the ways mWork impacts employee well-being. Ultimately, understanding the interplay between fixed and flexible factors is essential for effectively addressing stress in today’s rapidly evolving work environment.

Finally, future research should explore how gender and the number of children are associated with workplace performance from various perspectives. In particular, studies that incorporate the implications of this study’s findings in addressing critical societal issues, such as declining birth rates in South Korea and other countries, could provide meaningful contributions.

### 5.4. Conclusions

This study applies the COR theory to the workplace context, offering valuable insights into the negative effects of mWork. The findings indicate that mWork increases job stress, which in turn leads to employee sleep deprivation—a critical factor affecting organizational performance. Furthermore, the study highlights the moderating roles of gender and number of children, revealing that women and employees with more children are more likely to experience stronger negative impacts from mWork. These results underscore the importance of tailored organizational interventions based on gender and parental responsibilities.

By expanding the empirical application of COR theory, this research provides a deeper understanding of how resource loss and conservation are influenced by individual and contextual factors. From a practical perspective, the study proposes actionable strategies for managing job stress and sleep deprivation, such as implementing support policies tailored to gender and the number of children. These interventions have the potential to enhance both employee well-being and organizational performance. Ultimately, the findings offer valuable guidance for creating healthier, more productive workplaces and fostering sustainable organizational growth.

## Figures and Tables

**Table 1:** Means, Standard Deviations, Correlations, and Reliabilities.

Variable	Mean	SD	1	2	3	4	5	6	7	8	9
1. Age (years)	41.78	10.97	―								
2. Education	2.50	0.91	0.26	―							
3. Tenure	7.67	6.84	0.44 ***	0.01	―						
4. Marital status	0.55	0.50	0.47 ***	0.11	0.27 ***						
5. Gender	0.50	0.50	−0.09	−0.14 *	−0.12 *	―					
6. Number of children	0.85	0.96	0.63 ***	−0.03	0.34 ***	0.59 ***	−0.06				
7. mWork	2.45	1.05	0.06	0.21 ***	0.07	0.08	−0.05	−0.01	(0.92)		
8. Job stress	2.96	0.75	−0.07	0.00	0.07	−0.02	0.09	−0.08	0.23 ***	(0.85)	
9. Sleep deprivation	2.89	0.83	−0.03	−0.11 *	−0.02	−0.02	0.05	−0.05	0.13 *	0.25 ***	(0.83)

Notes: n = 325; * *p* < 0.05, *** *p* < 0.001 (two-tailed); the values in parentheses denote Cronbach’s alphas; Education = highest education level achieved: 1 = high school graduate, 2 = 2-year college graduate, 3 = 4-year university graduate, 4 = master’s graduate, 5 = Ph.D. holder; Tenure = organizational tenure (year); Gender, male = 0, female = 1; Number of children: no children = 0, 1 child = 1, 2 children = 2, 3 children = 3, over 4 children = 4.

**Table 2:** Confirmatory Factor Analysis Results.

Model	χ^2^ (df)	CFI	TLI	RMSEA	Δχ^2^ (Δdf)
Research model (3 factor)	201.35 (115) ***	0.97	0.96	0.05	
Alternative model 1 (2 factor) ^1^	892.67 (121) ***	0.69	0.63	0.14	691.32 (6) ***
Alternative model 2 (1 factor) ^2^	1263.06 (126) ***	0.55	0.47	0.17	1061.71 (11) ***

Notes: n = 325; *** *p* < 0.001; ^1^ Two-factor model with mWork and job stress on the same factor. ^2^ A one-factor model in which mWork, job stress, and sleep deprivation load onto a single latent construct. Abbreviations: CFI, comparative fit index; TLI, Tucker–Lewis index; RMSEA, root mean square error of approximation.

**Table 3:** Hierarchical Multiple Regression.

Variable	Job Stress			Sleep Deprivation		
	Model 1	Model 2	Model 3	Model 4	Model 5	Model 6
Age	−0.08	−0.10	−0.09	0.02	0.01	0.03
Education	0.01	−0.04	−0.05	−0.12 *	−0.15 *	−0.14
Tenure	0.14 *	0.13 *	0.14 *	−0.00	−0.01	−0.04
Marital Status	0.05	0.03	0.03	0.04	0.03	0.02
Gender (A)	0.10	0.10	0.11 *	0.03	0.03	0.02
Number of children (B)	−0.11	−0.08	−0.06	−0.10	−0.07	−0.05
mWork (C)		0.24 ***	0.26 ***		0.16 **	0.10
(A) × (C)			0.12 *			
(B) × (C)			0.12 *			
Job stress						0.23 ***
R^2^	0.03	0.09	0.11	0.02	0.04	0.09
ΔR^2^		0.06	0.02		0.02	0.05
adj R^2^	0.01	0.07	0.09	0.01	0.04	0.09
F	1.78	4.32 ***	4.39 ***	0.98	1.95 *	3.82 ***
F_inc_		18.95 ***	4.33 *		7.70 **	16.21 ***

Notes: n = 325; * *p* < 0.05; ** *p* < 0.01; *** *p* < 0.001 (two-tailed test). The results are standardized regression coefficients.

**Table 4:** Results of Bootstrapped Indirect Effect Test.

	Dependent Variable: Sleep Deprivation			
Mediating Variable	Indirect Effect	SE	95% CI	
			LLCI	ULCI
Job stress	0.04	0.01	0.01	0.07

Notes: n = 325, Number of bootstrapping iterations = 10,000. Abbreviations: SE, standard error; CI, confidence interval; LLCI, lower limit of confidence interval; ULCI, upper limit of confidence interval.

## Data Availability

The raw data supporting the conclusions of this article will be made available by the authors, without undue reservation, to any qualified researcher.

## Appendix B. Measurement

mWork (α = 0.92)

1. How frequently do you use a mobile device to perform your job during non-work hours?
2. To what extent do you use a mobile device to perform your job during non-work hours?
3. How frequently do you use a mobile device to handle some of your work demands during non-work hours?

Job stress (α = 0.85)

1. I experienced tension from my job.
2. Aspects of my job are a source of frustration for me.
3. There is no strain from working in my job.
4. I never feel pressured in my job (R).

Sleep deprivation (α = 0.83)

1. I had trouble falling asleep.
2. I had trouble staying asleep (including waking up too early).
3. I woke up several times during the night.
4. I woke up after obtaining my usual amount of sleep feeling tired and worn out.
